# Bottom Crack Detection with Real-Time Signal Amplitude Correction Using EMAT-PEC Composite Sensor

**DOI:** 10.3390/s24165196

**Published:** 2024-08-11

**Authors:** Yizhou Guo, Yu Hu, Kai Wang, Yini Song, Bo Feng, Yihua Kang, Zhaoqi Duan

**Affiliations:** School of Mechanical Science and Engineering, Huazhong University of Science and Technology, Wuhan 430074, China; m202270767@hust.edu.cn (Y.G.); huyuhy@hust.edu.cn (Y.H.); wang2kai@hust.edu.cn (K.W.); songyini@hust.edu.cn (Y.S.); bofeng@hust.edu.cn (B.F.); yihuakang@hust.edu.cn (Y.K.)

**Keywords:** bottom crack, lift-off, EMAT, PEC, amplitude correction, signal fusion

## Abstract

During electromagnetic ultrasonic testing, it is difficult to recognize small-size bottom cracks by time of flight (ToF), and the lift-off fluctuation of the probe affects the accuracy and consistency of the inspection results. In order to overcome the difficulty, a novel composite sensor of an electromagnetic acoustic transducer (EMAT) and pulse eddy current (PEC) is designed. We use the amplitude of a bottom echo recorded by EMAT to identify the tiny bottom crack as well as the amplitude of PEC signals picked up by the integrated symmetric coils to measure the average lift-off of the probe in real time. Firstly, the effects of lift-off and bottom cracks on the amplitude of bottom echo are distinguished by combining the theoretical analysis and finite element method (FEM). And then an amplitude correction method based on the fusion of EMAT and PEC signals is proposed to reduce the impact of lift-off on the defect signal. The experimental results demonstrate that the designed composite sensor can effectively detect a bottom crack as small as 0.1 mm × 0.3 mm. The signal fusion method can accurately correct the amplitude of defect signals and the relative error is less than ±8%.

## 1. Introduction

Crack emergence and expansion are among critical factors that affect the performance and longevity of structural steel such as plates, pipelines, and so on [1,2]. Typically, the cracks usually start with the opening sizes of 0.05–0.40 mm and may develop to dangerous sizes of tens of millimeters [1]. Non-destructive testing (NDT) techniques are used to inspect materials without damaging the sample under detection [3]. In NDT fields, the common methods of surface cracks detection mainly include Magnetic Flux Leakage (MFL) [4,5], Eddy Current Testing (ECT) [6,7,8], which are only able to detect the cracks near the surface due to the skin effect. Ultrasonic testing (UT), usually combined with phased-array systems, is capable of detecting cracks located deep in the sample, which requires both the coupling agent and the smooth surface [9,10,11]. EMAT is a new type of non-contact ultrasonic transducer, which has unique advantages in NDT and internal structure evaluation due to its outstanding advantages such not requiring contact or a coupling agent, its flexible generation of multiple acoustic modes, and its suitability for harsh environments such as high temperatures [12,13,14].

In electromagnetic ultrasonic inspection, different modes of ultrasonic waves are suitable for different inspection occasions. Ultrasonic-guided waves can be used for long-distance defect detection and structural health monitoring [15,16], while bulk waves can be used for identification, localization, and imaging of local defects [17,18]. The temporal technique is a commonly used method for bulk wave in pulsed-echo mode, in which a ultrasonic wave is excited in the testing sample by an EMAT, and the internal cracks can be assessed by the ToF of the defect echo [19]. However, some internal defects with relatively small sizes compared to the ultrasonic transducer, such as cracks, struggle to extract the defect echo because the energy of the ultrasonic wave reflected back from the defect wall is too low. In order to enhance the amplitude and signal-to-noise ratio (SNR) of the EMAT signals to improve the detection capability of defects, many researchers have implemented parameter optimization, signal processing and new probe designs [20,21,22,23]. However, in harsh industrial environments, the above methods are not effective, because the background industrial noise may cover up the defect echoes. Therefore, using the amplitude attenuation of a bottom echo to identify the tiny cracks based on inversion is more appropriate. Parra-Raad et al. designed an EMAT that could excite orthogonal shear waves and used it to identify and determine the direction of cracks through the amplitude difference [24]. But few researchers have investigated bottom crack defects with a width and depth of less than 1 mm by using EMAT, and our study makes up for this part and explains the rationality and advantages of using bottom echo amplitude detection in principle.

It is worth noting that in industrial inspection, the fluctuation of probe lift-off caused by vibration of the drive mechanism, non-conductive coatings, etc. can significantly affect the amplitude of detection signals and lead to confusion and misjudgment of defect signals [25,26]. Therefore, it is necessary to monitor the lift-off of probe in real time and reduce the effect of lift-off on the detection signal. Xiang et al. investigated the effect of lift-off on the frequency and peak amplitude of EMAT surface wave excitation process [27]. Hu et al. proposed a method on the basis of the permanent magnetic field perturbation (PMFP) theory to compensate for the defect signal using the measured lift-off information [28]. Duan et al. designed a new type of pulsed eddy current (PEC) probe array with different lift-offs and linearly combined the signal features under different lift-offs to reduce the effect of lift-off [29], and the method was extended to EMAT [30]. The amplitude attenuation of EMAT signals due to bottom defects follows the same trend caused by the increased lift-off. Therefore, it is necessary to distinguish their differences in principle and extract a signal feature that is only affected by lift-off rather than the defect based on the multi-physical fields coupling process of EMAT, which can be used to correct the effect of lift-off on crack detection signal.

To address this research gap, we propose a novel EMAT-PEC composite sensor consisting of a butterfly coil and a pair of rectangular coils. The amplitude of the EMAT signal recorded by the butterfly coil can be used to identify the tiny bottom cracks, and the PEC signals obtained by the integrated symmetrically arranged rectangular coils can be used to measure the average lift-off of the probe in real time during the scanning inspection. In order to eliminate pseudo-defect signals caused by probe jitter and to improve the accuracy of detection, we propose a multi-signal fusion method that can correct the amplitude of defect detection signal to reduce the effect of lift-off. Finally, we fabricate the composite sensor and probe for validating and applying the proposed method.

The other sections are organized as follows. Section 2 establishes a numerical theoretical model of the bulk wave EMAT and lift-off, distinguishes the effect of bottom crack and lift-off on the detection signal, and designs an EMAT-PEC composite sensor. Section 3 explores the fitting relationship between multiple signals recorded by the composite sensor and lift-off using the FEM and proposes a signal fusion method for PEC and EMAT signals to correct the amplitude of the defect detection signal. Section 4 focuses on the construction of an experimental platform and validation experiments to evaluate the performance of the proposed sensor and signal correction and fusion approach. Finally, Section 5 summarizes the innovations and contributions of the article and looks forward to future research directions.

## 2. Theory and Composite Sensor Design

### 2.1. Principle of Bulk Wave EMAT

**Figure 1:**
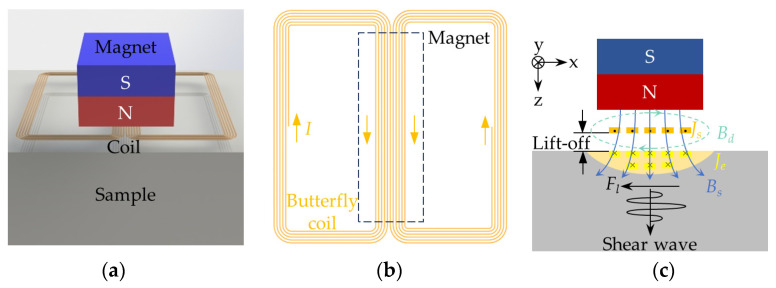
Schematic of the bulk wave EMAT: (**a**) Basic components of EMAT; (**b**) a butterfly coil; (**c**) the excitation process of shear wave based on the Lorentz mechanism.

A typical bulk wave EMAT consists of a permanent magnet, a coil, and a conductor sample, as shown in Figure 1a. Figure 1b shows a butterfly coil with equal spacing and the same direction in the central part. Compared to the spiral coil and racetrack coil, the butterfly coil excites shear wave with a smaller divergence angle and more intensive distribution, providing stronger detectability [31].

The excitation process of shear wave based on the Lorentz mechanism of EMAT is illustrated in Figure 1c. A coil fed with high-frequency alternating current *J_s_* is placed near the surface of the testing sample, which generates dynamic magnetic field *B_d_* in the surroundings and induces eddy current *J_e_* in the sample. Eddy current *J_e_* generates Lorentz force *F_l_* in interaction with dynamic magnetic field *B_d_* and biased static magnetic field *B_s_* provided by the permanent magnet, causing high-frequency vibrations of the particles inside the sample, which excites the shear waves. Based on Maxwell’s laws, the governing equations for the energy conversion process can be expressed as follows:

(1) $$\nabla \;\times \;H_{d}={\;J}_{s}\;,$$

(2) $$B_{d}={{\mu_{0}\;\mu }_{r}\;H}_{d}\;,$$

(3) $$\nabla \;\times \;E_{e}=-\frac{\partial B_{d}}{\partial t}\;,$$

(4) $$J_{e}=\sigma \;E_{e\;},$$

(5) $$F_{l}={\;J}_{e}\;\times \;(B_{d}+{\;B}_{s})\;\approx \;J_{e}\;\times \;B_{s}\;,$$

where *H_d_* denotes the dynamic magnetic field intensity, *μ*_0_ (= 4π × 10^−7^ H/m) and *μ_r_* denote the vacuum permeability and the relative permeability of the sample, respectively. *E_e_* denotes the electric field intensity and *σ* denotes the electrical conductivity of the sample. In Equation (5), the dynamic magnetic field *B_d_* can be neglected for a relatively small excitation pulse current [32].

The governing equation for the vibrations of the particles inside the sample induced by the Lorentz forces can be expressed as follows:

(6) $$G\nabla^{2}u+(G\;+\;\kappa )\;\nabla \;(\nabla \;\cdot \;u)+F_{l}=\rho \frac{\partial^{2}u}{\partial t^{2}},$$

where *G* and *κ* denote the Lamé constants of the material, *u* denotes the displacement of the particles inside the sample, and *ρ* denotes the density of the material.

The shear wave excited in pulse-echo mode propagates in the thickness direction, undergoes reflection and mode conversion at the bottom or defects, and then part of it propagates back to the surface. Based on the inverse Lorentz effect, the vibrations of the particles induced by the reflected shear wave generate eddy current *J_u_* under the interaction with biased static magnetic field *B_s_*, which is provided by the magnet. The dynamic magnetic field generated by *J_u_* passes the material–air boundary and is captured by EMAT. The induced eddy current *J_u_* can be expressed as follows:

(7) $$J_{u}=\sigma \;{(E}_{u}+\frac{\partial u}{\partial t}).$$

**Figure 2:**
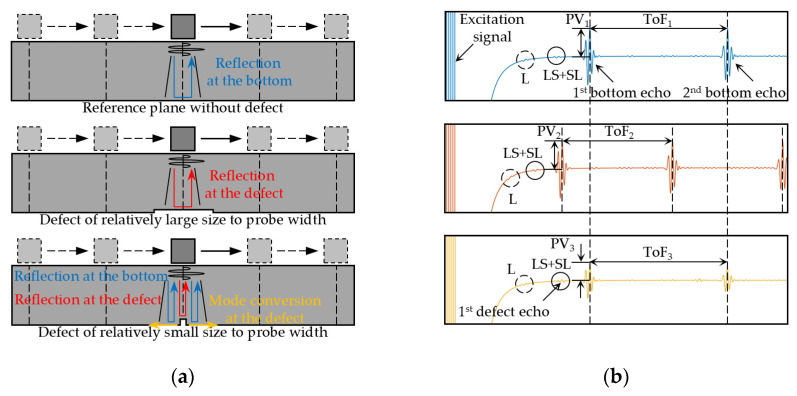
Pulse-echo mode for the bulk wave EMAT: (**a**) Reflection and mode conversion of ultrasonic wave at defects of different relative sizes to the probe width; (**b**) corresponding time-domain signals picked up by EMAT.

Figure 2a shows the reflection and mode conversion of shear wave occurring at defects of different relative sizes to the probe width. In this paper, the probe width is expressed as the width of the center region of the butterfly coil, as shown in Figure 1b. Figure 2b illustrates their corresponding time-domain detection signals picked up by EMAT.

1. When there are no bottom defects, the excited shear wave is reflected at the bottom, showing several echoes in the time-domain signal. This configuration of EMAT also excites less longitudinal wave, which induces reflection and mode conversion at the bottom to form reflected longitudinal waves, shear-longitudinal (SL), and longitudinal-shear (LS) converted waves. They are located between adjacent echoes due to their propagation speed, and are considered to be noise signals, affecting defect detection. The ToF_1_ of adjacent echoes can be used to evaluate the wall thickness of the sample.
2. When the bottom defect is relatively small compared to the probe width, the beam width of the shear wave excited by EMAT is smaller than the defect width, and a large portion is reflected by the defect, a changed ToF_2_ can be observed.
3. When the bottom defect, such as cracks, is relatively large compared to the probe width, the beam width is larger than the defect width, and only a small portion is reflected at the interface of the defect. The rest is reflected at the bottom or affected by the side of the defect to produce scattered waves in other directions, thus showing a changed peak value (PV) of the first bottom echo.

For the detection of small cracks, the amplitude of echo reflected by the defect is relatively small, which is not convenient to extract and is easily overlapped or confused with the converted waves described above. The amplitude of the bottom echo has a relatively large SNR, and the crack can be recognized and assessed by its attenuation. In combination with a scanning mechanism, the maximum attenuation of the echo amplitude can be used as the standard for the localization of crack.

Factors such as the coil design parameters and lift-off of the probe also affect the signal of EMAT, resulting in variations in its amplitude, so it is necessary to study the mechanism of their influence.

### 2.2. Effect of Lift-Off on Lorentz Force of Bulk Wave EMAT

**Figure 3:**
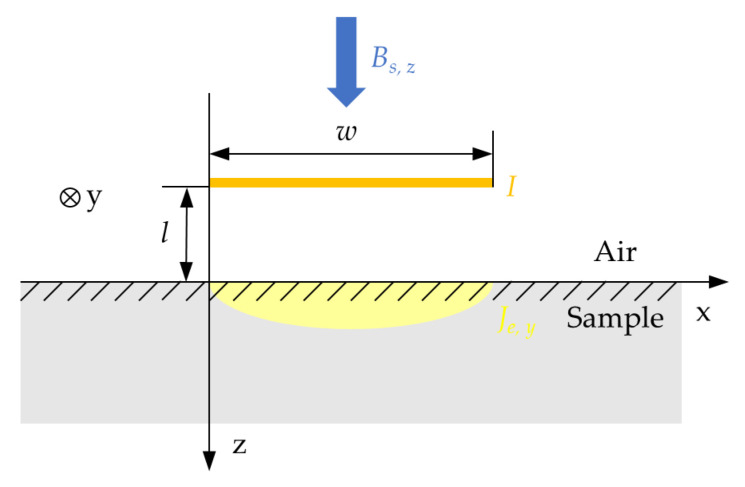
Two-dimensional simplified model of butterfly coil EMAT.

As shown in Figure 3, to simplify the analysis, the central working region of the butterfly coil is approximated as a thin current sheet of width *w* and the whole space is regarded as the existence of a constant and uniformly distributed magnetic field *B_d_*_,_*_z_*. The boundary between the sample and the air is set to be the x-axis, and the left edge of the current sheet is set to be the z-axis. *z* > 0 space is filled with the sample made of conductive material, and *z* < 0 space is filled with air. We assume that the turns per unit length of the coil are represented by *n* and the current flowing through the coil is *I*. In this two-dimensional approximate model, Equations (1)–(3) can be simplified as follows:

(8) $$\frac{{\partial H}_{x}^{S}}{\partial z}-\frac{{\partial H}_{z}^{S}}{\partial x}=J_{e,\;y},$$

(9) $$\frac{\partial E_{y}}{\partial z}=\mu_{0}\mu_{r}\frac{\partial H_{x}}{\partial t},$$

(10) $$\frac{\partial E_{y}}{\partial x}={-\mu }_{0}\mu_{r}\frac{\partial H_{z}}{\partial t}.$$

Combining Equations (4), (9) and (10), we obtain

(11) $$(\frac{\partial^{2}}{\partial x^{2}}+\frac{\partial^{2}}{\partial z^{2}}){\;H}_{x}-\;jw\sigma \mu_{0}\mu_{r}H_{x}=0.$$

In air, the $H_{x}^{A}$ satisfies

(12) $$(\frac{\partial^{2}}{\partial x^{2}}+\frac{\partial^{2}}{\partial z^{2}})\;H_{x}^{A}=0.$$

The tangential magnetic field at the origin (0,0) provided by the current element *nIdx* at (*x*,*z*) can be expressed as

(13) $$dH_{x}^{A}=\frac{\mathrm{nIdx}}{2\pi (x^{2}+z^{2})}\cdot \frac{2z}{\mu_{r}+1}.$$

We assume that the charge is uniformly distributed on the current sheet (*X*, −*l*), *X* ∈ (0, *w*), and *l* denotes the lift-off of the coil. At the boundary between the sample and the air, the tangential magnetic field satisfies the boundary conditions; thus, the tangential magnetic field at (*x*,0) provided by the entire current sheet can be expressed in the integral form of Equation (13) as follows

(14) $$H_{x}^{S}\left(x,0\right)=H_{x}^{A}\left(x,0\right)=\frac{\mathrm{nI}}{\pi (\mu_{r}+1)}\cdot \int_{0}^{w}\frac{[0-(-\;l)]\mathrm{dX}}{{\left(x-X\right)}^{2}+{[0-(-\;l)]}^{2}}=\frac{\mathrm{nI}}{\pi (\mu_{r}+1)}\cdot \;[\arctan\left(\frac{x}{l}\right)-\arctan\left(\frac{x-w}{l}\right)].$$

In sample, the *H_x_* satisfies

(15) $$\frac{{\partial^{2}H}_{x}^{S}(x,z)}{{\partial z}^{2}}-q^{2}H_{x}^{S}(x,z)=0,$$

where *q* = −(1 + *j*)/*δ*, *δ* denotes the skin depth of the sample

(16) $$\delta =\sqrt{\frac{2}{w\mu_{r}\mu_{0}\sigma }}.$$

Combining Equations (14) and (15), we obtain

(17) $$H_{x}^{S}\left(x,z\right)=\frac{\mathrm{nI}}{\pi (\mu_{r}+1)}\cdot e^{-(j+1)\frac{z}{\delta }}\cdot \;[\arctan\left(\frac{x}{l}\right)-\arctan\left(\frac{x-w}{l}\right)].$$

Substituting Equation (17) into Equation (8), we obtain

(18) $$J_{e,\;y}(x,z)=\frac{{\partial H}_{x}^{S}(x,z)}{\partial z}=\frac{\left(1\;+\;j\right)}{\delta }H_{x}^{S}(x,z).$$

The eddy current density inside the sample at *x* can be expressed as

(19) $$J_{e,\;y}(x)=\int_{0}^{\infty }J_{e,\;y}(x,z)\mathrm{dz}\propto [\arctan\left(\frac{x}{l}\right)-\arctan\left(\frac{x-w}{l}\right)].$$

**Figure 4:**
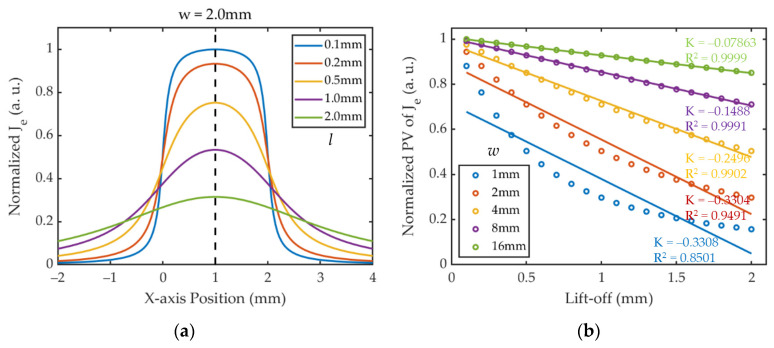
The results of numerical calculations from Equation (19): (**a**) The profiles of normalized *J_e_* under different *l*; (**b**) the curves of normalized PV of *J_e_* vs. lift-offs under different *w*.

Equation (19) shows that the distribution of *J_e_* is mainly determined by the width *w* and lift-off *l* together, provided that other parameters are constant. Assuming *w* = 2 mm, the profiles of normalized *J_e_* with different lift-offs are shown in Figure 4a. In the case of constant *w*, *J_e_* is symmetrically distributed about *x* = *w*/2, with the peak value occurring at that point, i.e., the center of the coil. And as *l* increases, *J_e_* decreases but the overall profile becomes wider. Assuming that the static magnetic field is constant and uniform, *F_l_* can be regarded as positively correlated with the magnitude of *J_e_* and can be considered to have the same spatial distribution as *J_e_*. Therefore, at a certain excitation frequency, the energy and radiation range of the shear wave excited by EMAT are mainly affected by the coil design parameter *w* and the lift-off distance *l*.

When *x* = *w*/2, Equation (19) can be expressed as

(20) $$J_{e,\;y}(\frac{w}{2})\propto 2\arctan\left(\frac{w}{2l}\right).$$

Equation (20) and Figure 4b show that the relationship between the PV of *J_e_* and *l* is consistent with an inverse tangent function. And in the same lift-off fluctuation range, as *w* increases, the absolute value of the slope for the curves also decreases, showing that coils with larger *w* have better resistance to lift-off interference. However, the coil with the larger width excites a wider beam of shear wave, resulting in lower sensitivity to cracks with small sizes, and therefore a compromise is needed for the parametric design of *w*.

The coil is affected by the change in the magnetic field in its surroundings and generates a corresponding voltage signal. The eddy current *J_e_* and the input excitation current *J_s_* contribute to the variation of the PEC signal, while the eddy current *J_u_* induced by the ultrasonic echo contributes to the variation in the EMAT signal. The lift-off fluctuation mainly affects the intensity of *J_e_*, which leads to an indirect effect on *J_u_*, causing changes in both EMAT and PEC signals, while the defect directly affects the intensity of *J_u_*, causing changes in the EMAT signal without affecting the PEC signal.

However, in industrial inspections, lift-off fluctuation is a non-negligible problem, and the EMAT signal is highly susceptible to lift-off, so it is necessary to design a sensor that can resist lift-off interference and to propose a method for real-time amplitude correction of EMAT signal during scanning detection.

### 2.3. EMAT-PEC Composite Sensor Design

**Figure 5:**
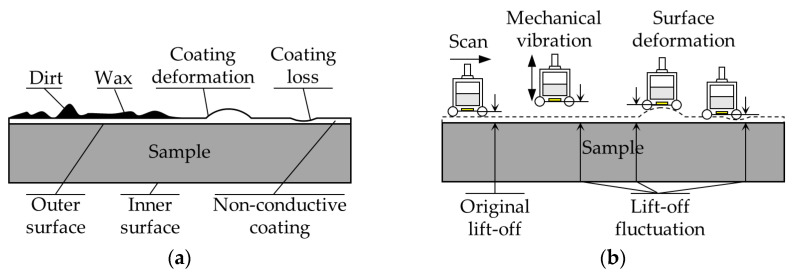
Schematic of the lift-off fluctuation in the scanning detection process: (**a**) Uneven outer surface of sample; (**b**) the lift-off fluctuations in several scenarios.

When the outer surface of sample is not smooth, there may be rough wax, attached dirt, coating loss, or coating deformation [25], as shown in Figure 5. The probe will be subjected to a reaction force perpendicular to the tangential direction of the outer surface, which can cause the probe vibration. Moreover, the probe is usually combined with the mechanical mechanism in a scanning inspection, and the random vibration of the machinery will also cause fluctuation of lift-off. All of the above will affect the accuracy and consistency of the inspection results.

Combining the analysis in Section 2.1 and Section 2.2, it can be concluded that the mechanisms of bottom crack and lift-off fluctuation resulting in the variation of EMAT signals are different. The former leads to the reflection, the scattering, and the mode conversion of the ultrasonic wave at the defect, resulting in the energy reduction in the ultrasonic echo. The latter affects the intensity of the induced eddy current during excitation, weakening the energy of the excited ultrasonic wave.

**Figure 6:**
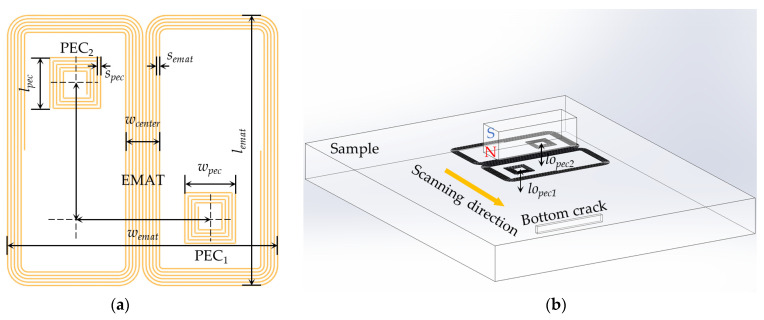
Schematic diagram of EMAT-PEC composite sensor: (**a**) Components of composite sensor; (**b**) side view of the whole probe.

In this paper, a compact EMAT-PEC composite sensor is designed with the structure shown in Figure 6. The composite sensor is center-symmetrical with a butterfly coil and two rectangular coils, which are located in the same plane. During each detection cycle, a pulse excitation is passed into the butterfly coil, and each rectangular coil senses the change in magnetic field in its surroundings to generate PEC signals. When the probe is operating smoothly, the waveform of the PEC signal is basically unchanged, and thus indicates that the density of the induced pulsed eddy current distributed on the surface of the sample is the same in each cycle, so it can be inferred that there is no change in the lift-off of the probe, and the variation in the amplitude of the EMAT signal can be used as a judgment for detecting the defect. When the probe undergoes lift-off fluctuation, the primary magnetic field generated by the excitation current does not cause a change in PEC signals because of the constant relative position between the EMAT coil and the PEC coils. But the lift-off fluctuation will affect the pulsed eddy current density and the secondary magnetic field generated by it, resulting in a change in the PEC signals. Therefore, extracting the PV of the PEC signals can characterize the lift-off information at the points of the symmetric rectangular coils and measure the average lift-off, which can be used to evaluate the variation in lift-off of the whole probe.

The proposed composite sensor has the following characteristics:

1. The butterfly coil can produce unidirectional and uniform shear wave with a small divergence angle, which has a good ability to detect tiny cracks perpendicular to the polarization direction;
2. The symmetric rectangular coils are used to measure the average lift-off of the probe. Compared to extracting PEC signals by a single coil or excitation coil, the use of symmetric rectangular coils can effectively improve the accuracy of the measurement in special industrial scenarios such as probe tilting and can achieve better applicability.
3. In each cycle, the PEC and EMAT signals originate from the same excitation signal, so it is easier to synchronize the acquisition of the PEC and EMAT signals and match them compared to the addition of other displacement sensors, which has positive implications for subsequent signal fusion and correction.
4. The proposed composite sensor uses only a single excitation signal to avoid crosstalk between multiple signals and improve the energy utilization of EMAT. The use of one transmitter and multiple receivers reduces the complexity of the circuit system, and the PEC signals can be extracted through a low-pass filter in the appropriate frequency band.

Based on the analysis in Section 2.2 and Figure 4b, we determined the *w_center_* to be 4 mm for the consideration of balancing the ability of tiny cracks detection and the resistance to lift-off fluctuation. The geometrical parameters of the composite sensor were optimized in our previous work [30], as shown in Table 1.

## 3. Simulation Analysis and Method

### 3.1. Finite Element Modeling

**Figure 7:**
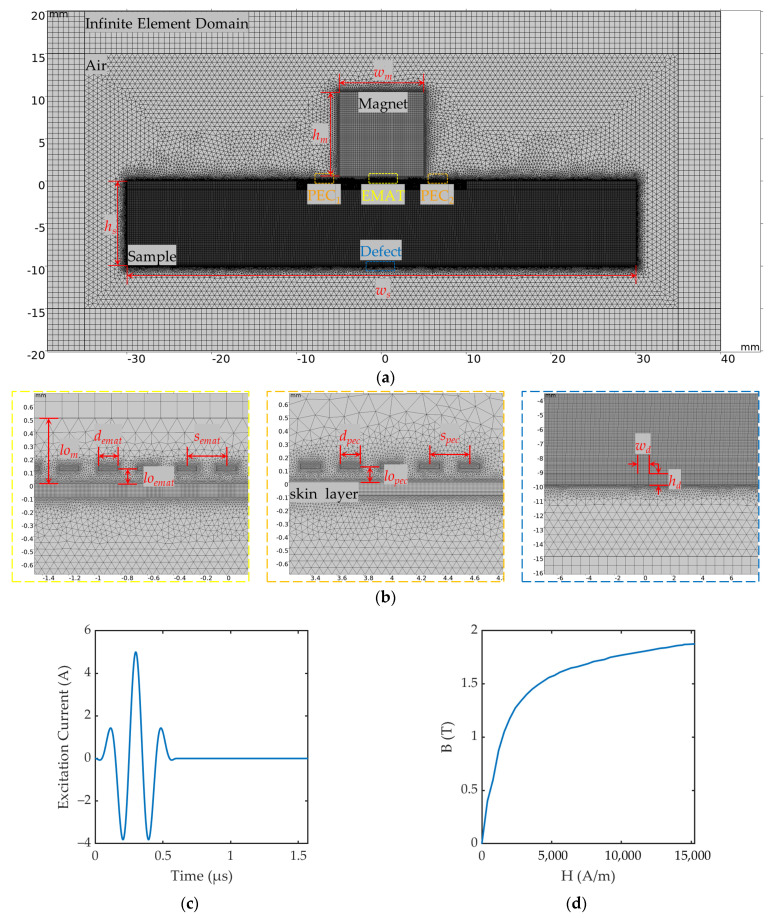
Schematic diagram of 2D FEM in COMSOL: (**a**) Overall view with mesh division; (**b**) partial enlarged views of the components; (**c**) excitation current of the EMAT; (**d**) B-H curve of the sample.

In order to further explore the ability of the proposed composite sensor to detect small-sized bottom cracks under the influence of lift-off fluctuation, a 2D FEM was established using COMSOL Multiphysics 6.2. The FEM was divided into six regions: permanent magnet, EMAT coil, PEC coils, sample, air, and infinite element domain, as shown in Figure 7a, and the additional parameters of the 2D FEM are shown in Table 2. The material of the permanent magnet was set as N52 NdFeB in the COMSOL material library, and the remanent flux density was set to 1.21T. The material of the coil was set to copper, with a density of 8960 kg/m^3^ and a conductivity of 5.998 × 10^7^ S/m. The excitation current injected into the coil consisted of a three-cycle tone-burst signal with a center frequency of 5 MHz modulated by the Hanning window, as indicated in Figure 7c. The material of the sample was set to be steel with a density of 7850 kg/m^3^, Young’s modulus of 200 × 10^9^ Pa, and Poisson’s ratio of 0.3. The electrical conductivity of the sample was 4.032 × 10^6^ S/m, and the permeability followed the B-H curve, as indicated in Figure 7d. The original lift-off of the coil was set to 0.1 mm and the original lift-off of the magnet was set to 0.5 mm. The infinite element domain was set up around the air to ensure the accuracy of the magnetic field of the permanent magnet at different lift-offs.

The Magnetic Fields component was set for the entire region of the FEM (finite element model) to calculate the static magnetic field generated by the permanent magnet, the dynamic magnetic field generated by the excitation signal passing through the coil, and the induced eddy current in the sample. The Solid Mechanics component was set for the sample region to calculate the generation and propagation of ultrasonic wave based on the Lorentz force mechanism. The regions of magnet, sample, and infinite element domain were mapped to reduce the complexity of calculation, and the regions of coils and air were meshed with Free Triangles to increase the flexibility of meshing in irregular area. It is worth noting that, to ensure the accuracy of eddy current calculations, a coupling region for the Lorentz force was set up on the upper surface of the sample region, which was two times thicker than the skin depth, and the maximum element size was 1/5 of the skin depth. The maximum element size for the rest of the sample was 1/10 of the wavelength of the ultrasonic wave. A Low-Reflection Boundary was set on both sides of the sample region to avoid ultrasonic waves reflected from the sidewalls affecting the subsequent acoustic field analysis. After building the mesh, the minimum element quality was 0.4558 and the average element quality was 0.9513.

To simulate the performance of the EMAT in pulse-echo mode for measuring bottom defect, the Transient Solver in COMSOL was employed to calculate the distributions of electromagnetic and acoustic fields for the time period of 0 to 2.4 × 10^−5^ s (1/*f*_c_ × 120) in steps of 1 × 10^−7^ s (1/*f*_c_/20). Meanwhile, we added Coil Geometry Analysis in FEM to directly obtain the EMAT and PEC signals picked up by the coils.

### 3.2. Crack Detection with Different Sizes

**Figure 8:**
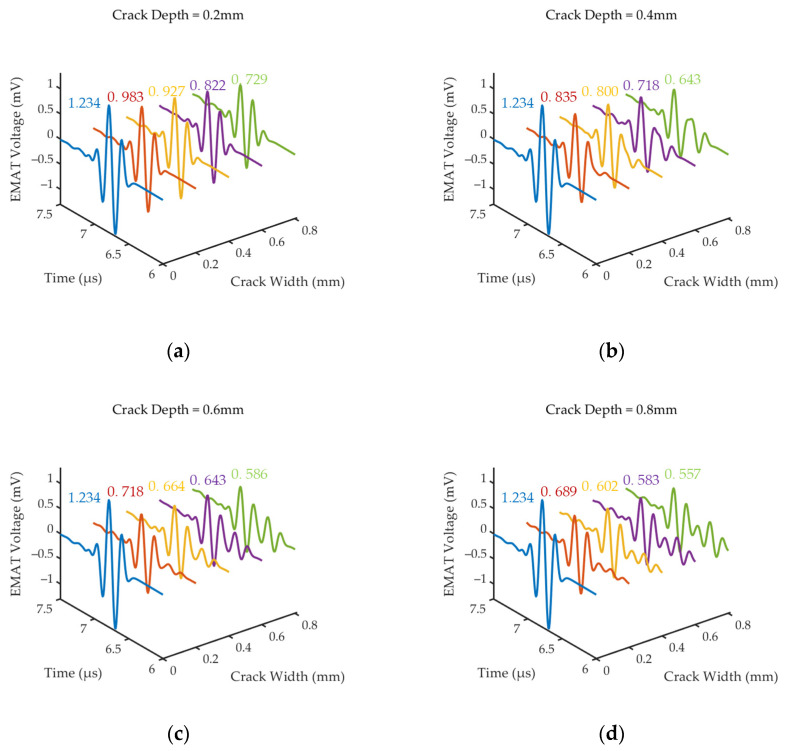
EMAT signals under cracks of different widths *w_d_* and depths *h_d_*: (**a**) Crack depth = 0.2 mm; (**b**) crack depth = 0.4 mm; (**c**) crack depth = 0.6 mm; (**d**) crack depth = 0.8 mm.

In order to study the effect of cracks with different depths and widths on the amplitude of a bottom echo recorded by EMAT, the rectangular area at the middle of the bottom of the steel sample was replaced with air to simulate the bottom crack, as shown in Figure 7b. Then, four groups of simulation models with various crack depths (0.2, 0.4, 0.6 and 0.8 mm) as well as for different crack widths (0.2, 0.4, 0.6 and 0.8 mm) were set up. Figure 8 shows the first bottom echo signals of EMAT, and the signal of ‘Crack width = 0.0 mm’ indicates the reference signal without defects. The simulation results show that the presence of a defect causes the attenuation of the bottom echo. And as the defect width and depth increase, the amplitude of the bottom echo decreases.

In Figure 8a–c, we can observe that the crack echo partially overlaps with the side flaps of the bottom echo, causing the signal features of the crack echo to be difficult to extract when the depth of the crack is relatively smaller than the wavelength (*λ* ≈ 0.64 mm). In Figure 8d, as the crack width increases, the amplitude of the crack echo increases. But even when the bottom echo amplitude decays to less than half of the reference signal amplitude, the crack echo amplitude fails to be equal to the bottom echo amplitude. Therefore, it is crucial to analyze the causes and understand the interaction of the shear wave with the crack at bottom.

**Figure 9:**
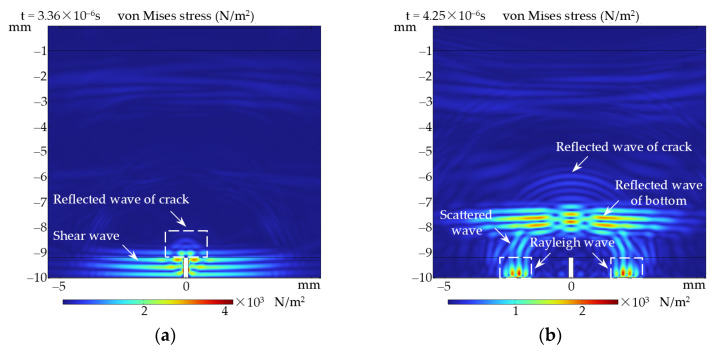
The simulation results of the acoustic field for the interaction of shear wave with the defect at the bottom: (**a**) T = 3.36 × 10^−6^ s; (**b**) T = 4.25 × 10^−6^ s.

Figure 9 illustrates the distributions of Von Mises stress inside the sample at two different moments for the detection of a crack with 0.8 mm depth and 0.2 mm width. Figure 9a shows that only a small portion of the incident shear wave polarized perpendicular to the crack is reflected at the interface of the crack, while the rest continues to propagate downward and interacts with both sides of the crack. Figure 9b shows that the shear wave scatters at the tips of the crack and undergoes a mode conversion with the interaction of the sides of the crack, generating Rayleigh waves [33]. Rayleigh waves propagate along the bottom of the sample to the sides, and will not be recorded by EMAT, resulting in further attenuation of the amplitude of the bottom echo signal. Moreover, it can be found that Rayleigh waves have a larger amplitude relative to the reflected wave of crack in Figure 9b, which explains why the attenuation of the bottom echo signal is larger than the amplitude of the crack echo signal.

Based on the above analysis, we can conclude that in the detection of cracks with small size, using the amplitude of the bottom echo as a signal feature to implement the detection has the following advantages:

1. The feature of the 1st bottom echo amplitude is easily extracted and the ToF between adjacent echoes can be used to synchronize the thickness measurement of the sample;
2. The amplitude of bottom echo is much larger than the amplitude of small-size crack echo, and a better SNR can be achieved;
3. Due to the complex reflection, scattering and mode conversion at crack-like defects, the crack echo is difficult to completely record using EMAT. Based on the inversion perspective, the amplitude attenuation of the bottom echo reflects the interaction of shear wave with the crack at bottom. The rate of change on bottom echo amplitude due to the crack is larger than on defect echo amplitude, and a higher detection sensitivity can be achieved.

### 3.3. Effect of Lift-Off on EMAT and PEC Signals

In Section 2.2, we consider the static magnetic field provided by the permanent magnet as a constant and assume that it is uniformly distributed in order to simplify the analysis. However, in the actual probe fabrication, the relative position between the permanent magnet and the coil is fixed, and when the lift-off of the coil is changed, the permanent magnet will change accordingly. In order to analyze the effect of lift-off on the EMAT and PEC signals and obtain accurate fitting relationships, which can be used to reduce the interference of probe jitter and lift-off fluctuation, we set up a group of simulation models with different lift-offs (*lo_ema_*_t_ = *lo_pec_* = {0.1, 0.2, 0.3, 0.4, 0.5, 1.0, 1.5, 2.0 mm}), as shown in Figure 7b, and the lift-off of magnet *lo_m_* is equal to *lo_ema_*_t_ plus 0.4 mm.

The skin depth of the steel sample calculated by Equation (16) is about 5.5 × 10^−6^ m, and thus the 2D cut line from point (−5 mm, −0.005 mm) to point (5 mm, −0.005 mm) is plotted in the datasets of the simulation results in order to analyze the distributions of *B_y_*, *J_e_*, and Von Mises stress in the energy-conversion region of EMAT.

**Figure 10:**
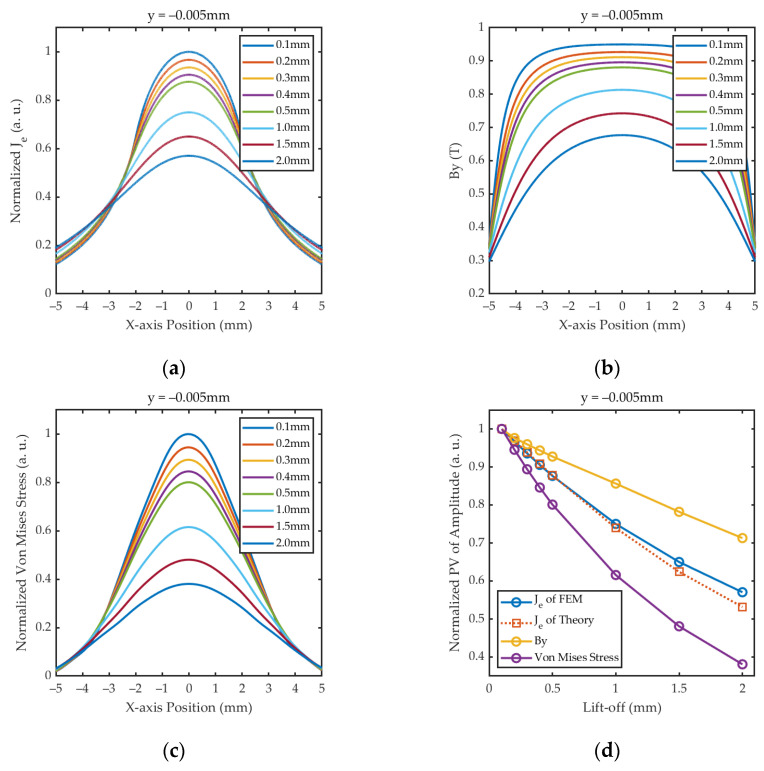
Simulation results of physical field distributions at 2D cut line between point (−5 mm, −0.005 mm) to point (5 mm, −0.005 mm) at 3 × 10^−7^ s under different lift-offs: (**a**) Distribution of *J_e_*; (**b**) distribution of *B_y_*; (**c**) distribution of Von Mises stress; (**d**) fitting curves of normalized PV of amplitude.

Figure 10a shows the simulation results of *J_e_*, which are in general agreement with the theoretical calculations in Section 2.2. Figure 10b,c show the distribution of magnetic field in the y-direction and Von Mises stress, respectively, and their amplitudes decrease with increasing lift-offs, which demonstrates that the lift-off fluctuation of the magnet further affects the accuracy of the detection results. Figure 10d illustrates that the fitting curve of *J_e_* from the FEM is shifted upwards from the theoretical calculation as the lift-off increases. The phenomenon can be explained by Equation (17), the FEM added to simulate the lift-off variation of the magnet, and based on the B-H curve of the ferromagnetic material, the relative permeability *μ_r_* decreases with the reduction in the applied magnetic field; thus, the simulated *J_e_* is slightly larger than the calculated value. Moreover, compared to the magnet, the lift-off fluctuation of the coil has a greater effect on the intensity of the ultrasonic wave, which severely decays when exposed to a combination of these two effects.

**Figure 11:**
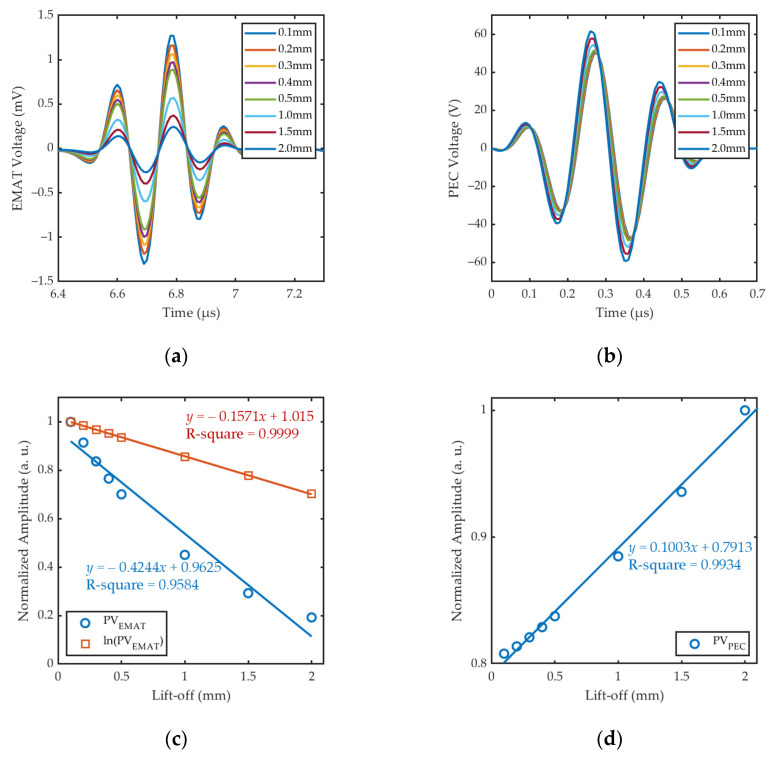
Simulation results of coil voltage under different lift-offs: (**a**) EMAT signals; (**b**) PEC signals; (**c**) comparison of the fitting curves of *PV*_EMAT_ and *ln*(*PV*_EMAT_); (**d**) fitting curve of *PV*_PEC_.

Based on Coil Geometry Analysis from COMSOL, we directly extracted the voltage signals of the EMAT coil and PEC coils under different lift-offs, as shown in Figure 11a and 11b, respectively. The voltage signals picked up by PEC_1_ and PEC_2_ coils are identical when the plane of composite sensor is horizontal. Figure 11c,d correspondingly illustrate the fitting curves of *PV*_EMAT_ and *PV*_PEC_ to lift-offs. With the designed coil parameters and configurations, the fitting curve of the *PV*_PEC_ is highly linear with the R-square of 0.9934. This is consistent with the theoretical prediction in Section 2.2 and *PV*_PEC_ can be used as an appropriate feature to measure the lift-off of the probe. The relationship between *PV*_PEC_ and lift-offs can be expressed as

(21) $${\mathrm{PV}}_{PEC1}=k_{1}\;\times \;{lo}_{1}+b_{1},\;{\mathrm{PV}}_{PEC2}=k_{2}\;\times \;{lo}_{2}+b_{2}.$$

The linearity of the fitting curve of *PV*_EMAT_ is poor, with an R-square of only 0.9584. After taking the logarithm of *PV*_EMAT_, the linearity of the fitting curve of ln(*PV*_EMAT_) and lift-offs is better with an R-square up to 0.9999, which is consistent with the study of [34]. The relationship between *PV*_EMAT_ and lift-offs can be expressed as follows:

(22) $${\mathrm{PV}}_{\mathrm{EMAT}}=b_{3}\times e^{-k_{3}\times {\;lo}_{3}}.$$

### 3.4. Real-Time Signal Correction and Fusion Method

**Figure 12:**
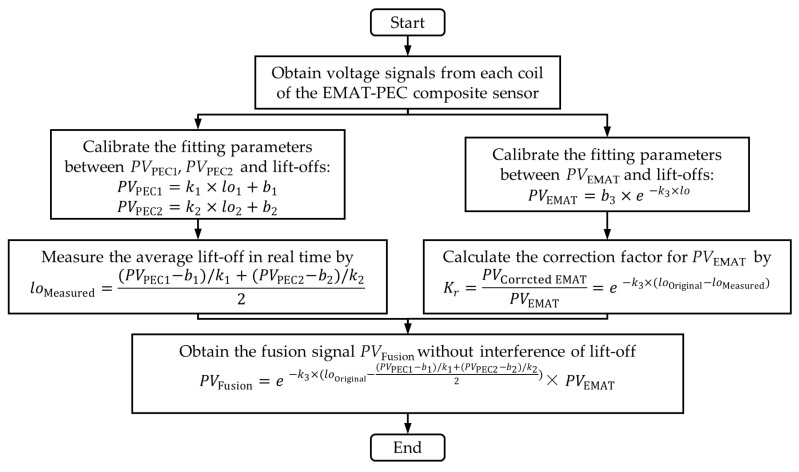
The flowchart of the signal correction and fusion under lift-off fluctuation.

In Section 3.3, we observe the fitting relationships between the two detection signals and the lift-offs extracted by the composite sensors. Subsequently, we propose a real-time detection signal correction and fusion method. The flowchart of signal correction and fusion is shown in Figure 12. The specific steps are as follows:

1. Step 1 is calibration. At the reference plane of the tested sample, a set of EMAT signals and PEC signals under the known lift-offs are extracted using the composite sensor to plot the fitting curves between each signal and lift-off and determine the fitting parameters.
2. Step 2 is the measurement of lift-off. During the crack detection, the peak value of PEC signals *PV*_PEC1_ and *PV*_PEC2_ picked up by PEC_1_ and PEC_2_ coils are substituted into the calibrated fitting curves to calculate the average lift-off of the composite sensor in real time.
3. Step 3 is the correction of detection signals. Based on the fitting curve between EMAT signal and lift-off, the formula for the signal correction factor can be obtained, and then the measured lift-off is substituted into the formula to calculate the value of the correction factor, and finally the correction factor is multiplied by *PV*_EMAT_ to obtain the crack detection signal without the interference of lift-off.
4. Step 4 is the fusion of detection signals. Combining the measurement and correction processes of steps 2 and 3, we can fuse the *PV*_PEC1_, *PV*_PEC1_, and *PV*_EMAT_ signals to form a new fusion signal which is unaffected by lift-offs.

The proposed composite detection method utilizes the lift-off measured using the PEC method to correct the detection signal of EMAT, compensating for the effect of lift-off fluctuation in the process of crack detection, which allows for a consistent assessment of the defect size inversion under different lift-offs. It should be noted that, in practice, it may happen that the lift-off is not uniform on both sides of the sensor, and therefore the average lift-off measured at two points by the centrally symmetric PEC coils is used as the equivalent lift-off for EMAT coil.

## 4. Experiment

### 4.1. Experimental Setup

**Figure 13:**
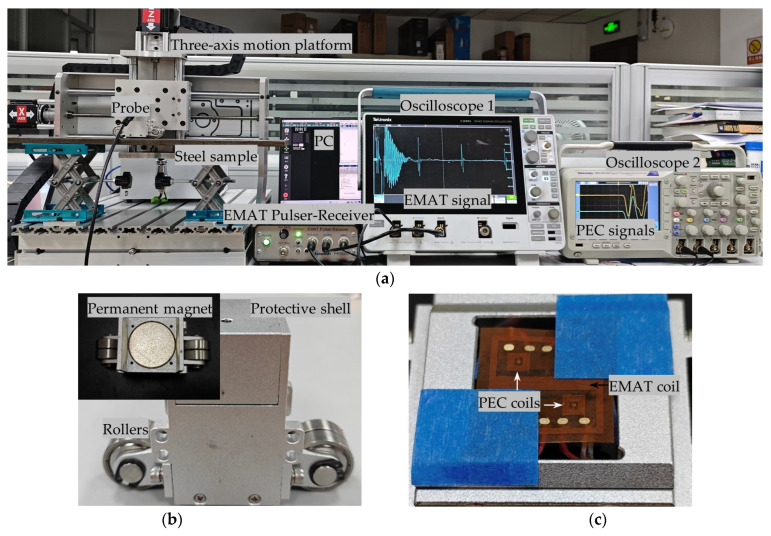
Experimental system of EMAT-PEC composite detection: (**a**) Experimental platform setup; (**b**) probe structure; (**c**) proposed EMAT-PEC symmetric composite sensor.

In order to validate the detection performance of the proposed composite sensor, the composite sensor was fabricated and the experimental system of EMAT-PEC composite detection was established, as shown in Figure 13. The EMAT-PEC composite sensor was excited by a PR5000 EMAT Pulser-Receiver (sonemaT, Coventry, UK), and the details of the instrument’s parameters and performance are described in [14]. The butterfly coil was directly connected to the PR5000 and served as both the excitation and receiving coil for EMAT. The received EMAT signal was filtered and amplified by the instrument and displayed the waveforms in oscilloscope 1. The symmetrically arranged rectangular coils served as the receiving coil of the PEC signals and connected to the oscilloscope 2 to display the waveforms after being filtered by the low-pass filter. The composite detection coils were fixed in an epoxy wear-resistant layer with a thickness of 0.2 mm, i.e., the original lift-off of the probe was determined to be 0.2 mm. The original lift-off of the permanent magnet was 1.0 mm, and the area between the detection coil and the magnet was filled with a copper foil to avoid interference caused by the eddy current generated inside the permanent magnet. A pair of rollers were set on both sides of the probe to minimize friction during scanning detection. The probe was fixed on a three-axis motion platform, which could drive the probe along the x-axis and y-axis for scanning detection and along the z-axis to simulate lift-off fluctuation. PC was used to control the three-axis motion platform while analyzing and processing the detection signals. The other parameters of the composite sensor are consistent with the parameters of FEM in Section 3.

**Figure 14:**
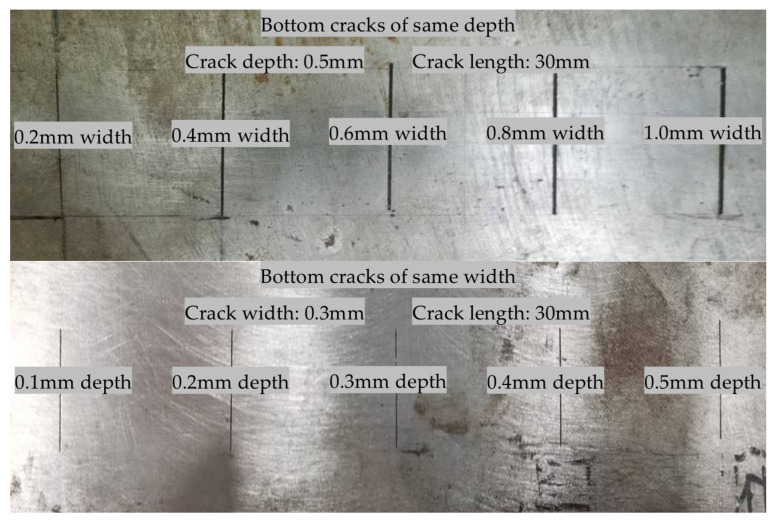
Size of manually machined different cracks on the bottom of the steel plates.

One side of the steel plates was manually machined with cracks of different sizes, as shown in Figure 14. The material of the first steel plate is 45 steel, and the size of it is 100 × 500 × 10 mm. The material of the second steel plate is Q235 steel, and the size of it is 100 × 500 × 20 mm. During the experiments, the probe was placed on the front side of the steel plates, and the cracks were distributed on the back side of the steel plates.

### 4.2. Results and Discussion

#### 4.2.1. Detection of Bottom Cracks at Different Widths

**Figure 15:**
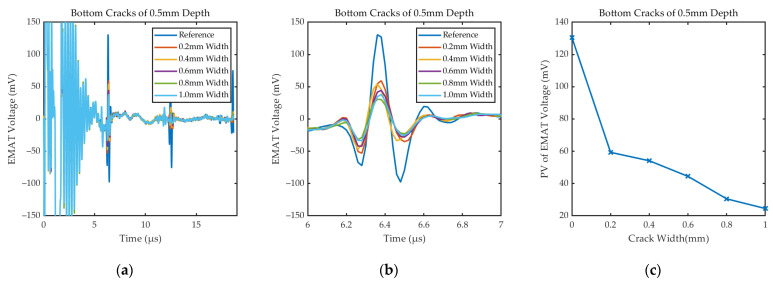
Signals received by EMAT under the cracks of different widths: (**a**) Specific waveforms; (**b**) the first bottom echo; (**c**) *PV*_EMAT_ under the cracks of different widths.

Figure 15 shows the specific waveforms and *PV*_EMAT_ for cracks of different widths. The experimental results show that the signal amplitude decreases with the increase in defect width, which is consistent with the law of the simulation results in Section 3.2. For cracks as small as 0.2 mm in width, the sensor still has good detection capability. The defect echo can be observed in Figure 15a, but its amplitude and rate of change are smaller than the bottom echo, which confirms the analysis in Section 3.2.

#### 4.2.2. Detection of Bottom Cracks at Different Depths

**Figure 16:**
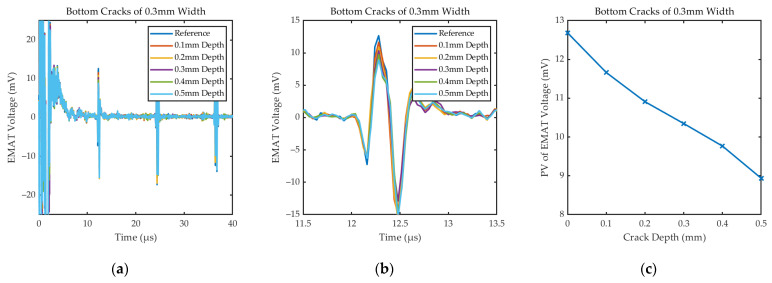
Received signals of EMAT under the cracks of different depths: (**a**) Specific waveforms; (**b**) the first bottom echo; (**c**) *PV*_EMAT_ under cracks of different depths.

Figure 16 shows the specific waveforms and *PV*_EMAT_ under the cracks of different depths. The experimental results show that the signal amplitude decreases with the increase in defect depth, which is consistent with the law of the simulation results in Section 3.2. Due to the different materials and thicknesses of the two steel plates, there are differences in the waveforms of their detection signals. For cracks as small as 0.1 mm in depth, the sensor is able to recognize changes in signal amplitude. However, the defect echo is virtually unobservable when the sample is very thick.

#### 4.2.3. Calibration Experiments

At the beginning of the experiment, the probe was placed flat on top of the steel plate for an original lift-off of 0.2 mm, then the z-axis of the motion platform was operated to elevate the probe from 0.2 mm to 2.0 mm at 0.2 mm intervals. The calibration experiments were repeated five times and the average peak value of the signals was obtained and recorded.

**Figure 17:**
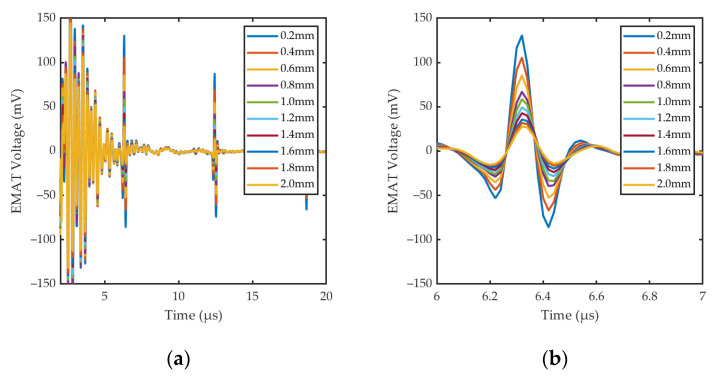

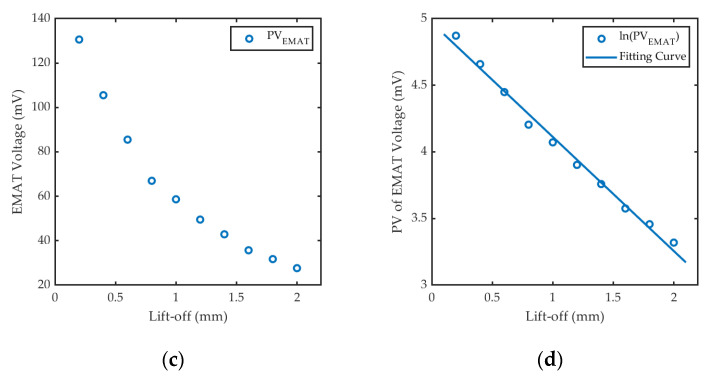
Received signals of EMAT coil under different lift-offs: (**a**) Specific waveforms; (**b**) the first bottom echo; (**c**) PV of EMAT voltage; (**d**) fitting curve between ln(*PV*_EMAT_) and lift-offs.

Figure 17a shows specific waveforms of EMAT voltage under different lift-offs. The relationships and fitting results of *PV*_EMAT_ and ln(*PV*_EMAT_) to lift-offs are shown in Figure 17b and c, respectively. The linearity of the fitting results between ln(*PV*_EMAT_) and lift-off obtained from the experiments is good, with an R-square greater than 0.99, which is consistent with the simulation results in Section 3.3.

**Figure 18:**
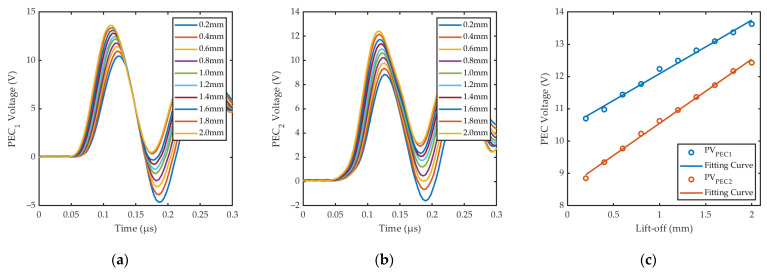
Received signals of symmetric PEC coils under different lift-offs: (**a**) Specific waveforms of PEC_1_; (**b**) specific waveforms of PEC_2_; (**c**) fitting curve between *PV*_PEC_ and lift-offs.

Figure 18a,b show the PEC signals received by the symmetric PEC coils under different lift-offs. The fitting results of *PV*_PEC_ and lift-offs are shown in Figure 18c. The linearity of the experimentally acquired fitting curves is good, with R-square greater than 0.99, which also matches the simulation results in Section 3.3. The signal waveforms of the two PEC coils are similar, but the amplitudes are different, which is due to the impedance difference between the symmetric PEC coils. It can be solved by increasing circuit components to match the impedance of PEC coils or using software for parameter adjustment to make two fitting curves consistent. In this paper, based on the existing experimental conditions, the two fitting curves obtained by calibration are used to measure the lift-offs at two points and calculate the average lift-off, which has no significant effect on the detection results.

The fitting functions of the signals obtained from the calibration experiments are shown in Table 3. Based on the relationship between multiple signal features and lift-offs, we are able to further develop lift-off measurement, signal fusion, and correction experiments.

#### 4.2.4. Comparison of Lift-Off Measurement between Symmetric PEC Coils and Single PEC Coil

**Figure 19:**
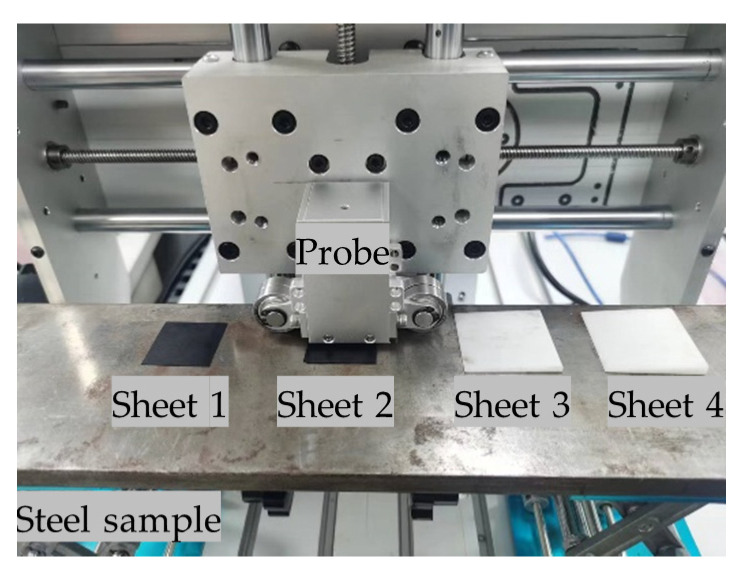
The setup of different sheets used to simulate lift-off fluctuation.

To demonstrate the effectiveness of the proposed lift-off measurement method and to check its accuracy, the sheets of different sizes shown in Figure 19 were set to simulate the lift-offs caused by the vibrations of the mechanism and the non-conductive coatings on the surface of the sample during the scanning detection. The thicknesses of sheet 1 and 2 are uniform and the measured actual thicknesses are 0.25 mm and 0.504 mm, respectively. The thicknesses of sheets 3 and 4 are uneven; the measured average thicknesses are 0.702 mm and 1.205 mm, respectively. Sheets 1, 2, 3, and 4 were set below the probe, and the *PV*_PEC1_ and *PV*_PEC2_ were recorded by the symmetric PEC coils and the average lift-off was calculated according to the fitting function in Table 3. The results are shown in Table 4.

From the experimental results, it can be seen that the utilization of symmetric PEC coils effectively measures the average lift-off of the probe, and the relative error is kept within ±5%. When the lift-offs on both sides of the probe are not consistent, the error of lift-off measurement is greater than 20% using only the PEC_1_ coil, while the design of the symmetric coil solves this problem.

#### 4.2.5. Amplitude Correction Experiment Based on Signal Fusion

Based on the signal correction and fusion method proposed in Section 3.3 and the fitting relationships obtained from the calibration experiments in Table 4, the signal after correcting, which is not affected by lift-off, can be represented the fusion of multiple signals, as follows:

(23) $${\mathrm{PV}}_{\mathrm{Fusion}\;}={e\;}^{-0.8565\;\times \;(0.2\;-\;\frac{{(\mathrm{PV}}_{PEC1\;}-\;10.43)\;/\;1.659\;+\;{(\mathrm{PV}}_{PEC2}-\;1.987)\;/\;8.564}{2})\;}\;\times \;{\mathrm{PV}}_{\mathrm{EMAT}}$$

**Figure 20:**
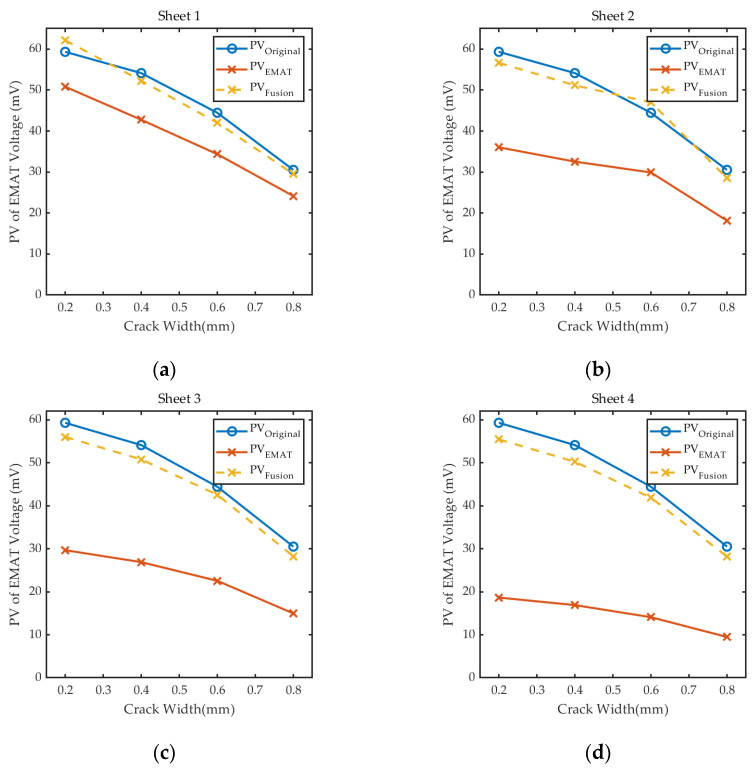
Correction results for crack detection signals under different lift-offs: (**a**) Lift-off caused by Sheet 1; (**b**) lift-off caused by Sheet 2; (**c**) lift-off caused by Sheet 3; (**d**) lift-off caused by Sheet 4.

In order to verify the effectiveness of the proposed real-time signal amplitude correction method based on the signal fusion, experiments were carried out to detect bottom cracks. We placed the sheets used in Section 4.2.4 under the probe to implement the detection. The *PV*_EMAT_ recorded by the EMAT coil and *PV*_Fusion_ calculated by Equation (23) are shown in Figure 20; *PV*_Original_ is the reference signal of the experimental results in Section 4.2.1. The specific experimental data for the detection and the results of signal correction are listed in Table 5.

The experimental results show that the composite detection sensor designed in this paper and the proposed real-time signal correction and fusion method are able to correct the abnormal defect detection signal, so that it is basically restored to the amplitude of the detection signal under the original lift-off. The relative error between the *PV*_Fusion_ and *PV*_Original_ is less than ±8%. Thus, it can be concluded that the method can reduce the effect of lift-off on the bottom defect detection signal and ensure the consistency and accuracy of the signal in the detection process.

## 5. Conclusions and Future Work

### 5.1. Conclusions

To identify tiny bottom cracks and to ensure the accuracy and consistency of the inspection results, this paper introduces a compact EMAT-PEC composite sensor which can simultaneously detect the bottom cracks via the amplitude attenuation of the EMAT bottom echo and evaluate the posture of the probe by the symmetric PEC signals. Based on the relationships between PEC and EMAT signals and lift-offs, a signal fusion method is proposed to correct the detection signal and reduce the lift-off effect. In comparison with traditional and single EMAT, the proposed composite sensor has three main advantages:

1. The optimally designed and arranged probe is capable of exciting unidirectional shear waves with a polarization direction perpendicular to the cracks, and the mode conversion occurring at the tiny crack causes the attenuation of the bottom echo amplitude. Compared with focusing on the defect echo, extracting the attenuation of the bottom echo amplitude to invert the defect size can provide a better SNR and resolution. The experimental results prove that the probe has the ability to detect bottom cracks as small as 0.1 × 0.3 mm;
2. The designed composite sensor can synchronously obtain the information of defects in a testing sample and probe posture in real time based on the EMAT echo signal and PEC signals. The whole system is supported by only one excitation source, which ensures the stability of the system and improves the energy utilization rate. The experimental results validate that, symmetrically, PEC coils can more accurately measure the average lift-off of the probe than a single PEC coil, with a relative error of less than ±5%.
3. The proposed signal fusion method skillfully combines the fitting relationships between the EMAT, PEC signals, and the lift-offs to form a fusion signal that is not affected by lift-off, thereby effectively reducing the impact of probe fluctuation on defect detection during actual inspection. The experimental results confirm that the signal fusion method effectively corrects the effect of lift-off on the amplitude of the detection signal, and the error of the corrected signal relative to the reference signal is less than ±8%.

### 5.2. Future Work

It is worth noting that the amplitude of the reflected shear wave strongly depends on the relative orientation of the cracks with respect to the direction of polarization of the incident shear wave. In this paper, we focus on the identification of tiny bottom cracks and the correction of the signal amplitude affected by lift-off fluctuation; therefore, we ignore the effect of the crack direction and set the scanning direction of the probe to be perpendicular to the direction of the cracks and the direction of the current in the excitation coil to be parallel to the direction of the cracks by default to obtain the best signal characteristics. In practice, the crack directions are varied, and it has been proposed to discriminate the direction and angle of the cracks by the amplitude ratio of two orthogonally arranged coils in [24]. The signal correction and fusion method proposed in our study is also of practical significance to improve the accuracy of the amplitude detection results of the two orthogonal coils.

Our future work will focus on designing a self-rotating probe to detect the direction of cracks. Subsequently, we will combine it with a scanning mechanism to form the c-scan image of the tiny bottom cracks. The signal fusion method proposed in this paper can be used for image correction during the probe rotation and scanning process.

## Figures and Tables

**Table 1:** Parameters of the EMAT-PEC symmetric composite sensor.

Object	Parameter	Symbol	Value
EMAT	Width	*w_emat_*	16 mm
	Length	*l_emat_*	16 mm
	Lift-off	*lo_emat_*	0.1 mm
	Wire diameter	*d_emat_*	0.2 mm
	Wire spacing	*s_emat_*	0.4 mm
	Wire height	*h_emat_*	0.035 mm
	Turns	*n_emat_*	11
PEC_1_, PEC_2_	Width	*w_pec_*	4 mm
	Length	*l_pec_*	4 mm
	Lift-off	*lo_pec_*	0.1 mm
	Wire diameter	*d_pec_*	0.2 mm
	Wire spacing	*d_pec_*	0.4 mm
	Wire height	*h_pec_*	0.035 mm
	Turns	*n_pec_*	5

**Table 2:** Additional parameters of 2D FEM.

Object	Parameter	Symbol	Value
Magnet	Width	*w_m_*	10 mm
	Height	*h_m_*	10 mm
	Lift-off	*lo_m_*	0.5 mm
	Remanent flux density	*B_s_*	1.21T
Sample	Width	*w_s_*	60 mm
	Thickness	*h_s_*	10 mm
	Density	*ρ*	7850 kg/m³
	Permeability	*μ*	B-H curve
	Electrical conductivity	*σ*	4.032 × 10^6^ S/m
	Young’s modulus	*E*	200 × 10^9^ Pa
	Passion’s ratio	ε	0.33
excitation current	Center frequency	*f_c_*	5 MHz
	Cycle	*n*	3

**Table 3:** Fitting function of calibration experimental results.

Fitting Function	R-Square
ln(*PV*_EMAT_) = −0.8565 × *lo* + 4.969	0.9911
*PV*_PEC1_ = 1.659 × *lo* + 10.43	0.9929
*PV*_PEC2_ = 1.987 × *lo* + 8.564	0.9971

**Table 4:** Comparison of measured lift-offs and actual lift-offs.

Sheet No.	*PV*_PEC1_ (V)	*lo*_1_ (mm)	*PV*_PEC2_ (V)	*lo*_2_ (mm)	Average *lo* (mm)	Actual *lo* (mm)	Relative Error
Sheet 1	11.138	0.427	9.444	0.443	0.435	0.250 + 0.2	−3.33%
Sheet 2	11.615	0.714	10.038	0.742	0.728	0.504 + 0.2	3.41%
Sheet 3	11.784	0.816	10.686	1.068	0.942	0.702 + 0.2	4.32%
Sheet 4	12.124	1.021	12.385	1.923	1.472	1.205 + 0.2	4.77%

**Table 5:** Experimental data and the results of signal correction.

Sheet No.	Crack Width (mm)	*PV*_EMAT_ (mV)	*PV*_Fusion_ (mV)	*PV*_Original_ (mV)	Relative Error
Sheet 1	0.2	50.809	62.138	59.327	+4.74%
	0.4	42.747	52.278	54.135	−3.43%
	0.6	34.361	42.022	44.455	−5.47%
	0.8	24.142	29.525	30.517	−3.25%
Sheet 2	0.2	36.034	56.639	59.327	−4.53%
	0.4	32.538	51.144	54.135	−5.53%
	0.6	29.900	46.997	44.455	+5.72%
	0.8	18.139	28.511	30.517	−6.57%
Sheet 3	0.2	29.685	56.046	59.327	−5.53%
	0.4	26.909	50.804	54.135	−6.15%
	0.6	22.556	42.586	44.455	−4.20%
	0.8	14.970	28.263	30.517	−7.39%
Sheet 4	0.2	18.660	55.470	59.327	−6.50%
	0.4	16.908	50.262	54.135	−7.15%
	0.6	14.093	41.894	44.455	−5.76%
	0.8	9.4888	28.207	30.517	−7.57%

## Data Availability

The raw data supporting the conclusions of this article will be made available by the authors on request.
